# Structured surgical training in minimally invasive esophagectomy (MIE) increases textbook outcome–a risk-adjusted learning curve

**DOI:** 10.1007/s00464-025-11539-1

**Published:** 2025-01-28

**Authors:** Philippa Seika, Friederike Martin, Armanda Serwah, Max Magnus Maurer, Axel Winter, Ramin Raul Ossami-Saidy, Paul V. Ritschl, Eva Dobrindt, Annika Kurreck, Jonas Raakow, Johann Pratschke, Matthias Biebl, Christian Denecke

**Affiliations:** 1 Department of Surgery, Campus Charité Mitte Campus Virchow-Klinikum, Charité Universitätsmedizin Berlin, Berlin, Germany; 2 Division of Gastroenterology, Department of Medicine, Beth Israel Deaconess Medical Center, Harvard Medical School, Boston, MA 02215 USA; 3 Division of Transplant Surgery, Department of Surgery, Brigham and Women´S Hospital, Harvard Medical School, Boston, MA 02215 USA; 4 Berlin Institute of Health, Charité Universitätsmedizin Berlin, Berlin, Germany; 5 Medizinische Klinik Mit Schwerpunkt Hämatologie, Onkologie Und Tumorimmunologie, Charité Universitätsmedizin, Campus Charité Mitte, Campus Virchow-Klinikum, 10117 Berlin, Germany; 6 Department of Surgery, Ordensklinikum Linz Barmherzige Schwestern, 4010 Linz, Austria

**Keywords:** Minimally invasive Ivor Lewis esophagectomy, Learning curve, Esophageal cancer

## Abstract

**Background:**

Minimally Invasive Esophagectomy (MIE) is a complex surgical procedure that has become a cornerstone in the management of esophageal cancer. This study aims to delineate the learning curve associated with MIE and its impact on patient outcomes.

**Methods:**

A retrospective analysis was conducted on 191 patients who underwent MIE between 2015 and 2022. The cohort was divided into two groups according to the level of competence: Trainer (*n* = 100) and Trainee (*n* = 91). Patient demographics, tumor characteristics, and surgical parameters were examined. RA-CUSUM methodology was employed to monitor patient outcomes, adjusting for variations in risk profiles using varying-coefficient logistic regression models to establish the MIE proficiency learning curve.

**Results:**

The trainee achieved competence in terms of operative time within 47 cases, following risk adjustment. Similarly, the learning curve in terms of major complications was completed after the 55th consecutive case. The LC was completed in terms of increased incidence of TO achievement in the trainee group after 83 cases (Trainer vs. Trainee, 27.00% vs. 40.66%, *p* = 0.046). Anastomotic leakage (Trainer vs. Trainee, 10.00% vs. 7.69% (*p* = 0.575)) could be identified with consistent rates for both trainer and trainee during the observational period. Pulmonary complications accounted for the majority of complications. After a follow-up of 2 years, no effect of the learning curve on overall (*p* = 0.436) or disease-free (*p* = 0.305) survival could be concluded, indicating consistent quality and patient safety during the surgical training.

**Conclusions:**

While technical competence can be achieved after approximately 55 cases, achievement of 'textbook outcome' (TO) requires 83 cases. The findings demonstrate that structured surgical training can progress in tandem while maintaining oncological safety for patients. While technical competence is crucial, the ultimate goal should be achieving a TO.

**Graphical Abstract:**

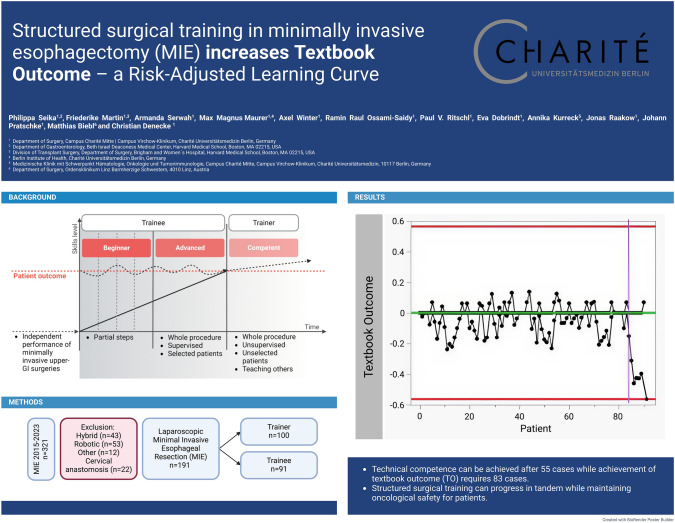

Esophagectomy is a cornerstone in the treatment of patients with esophageal cancer (EC) and cancer of the gastroesophageal junction (CGEJ). For a long time, open esophagectomy was the only surgical treatment for patients with resectable EC/CGEJ [1]. However, in the last decades, growing laparoscopic experiences led to the successful introduction of minimally invasive techniques for esophagectomy [2]. While minimally invasive esophagectomy (MIE) reduced the risk for postoperative morbidity compared to an open approach, it presents as a highly challenging technique for both the performing surgeons and the surrounding teams [3–5]. The acquisition of the necessary surgical skills for such a procedure inevitably bears the risk of increased morbidity, mortality, and compromised oncological outcomes during the learning period [6, 7]. Therefore, it is critical to keep the learning curve (LC) for surgeons as steep and short as possible so that the procedure´s outcome advantages come to the fore [8]. This is relevant not only in terms of the time for implementation of this new technique, but also to maintain quality and patient safety in the long term after its successful implementation. To guarantee this, surgeons must be trained properly to perform this demanding technique safely while also obtaining the necessary skills quickly. To ensure short LCs and to keep risks for patients as low as possible during the learning process, standardized teaching/learning concepts including thorough patient selection and quality control are therefore mandatory.

In our surgical department, which comprises two campuses and is accredited as a tertiary referral center for gastroesophageal surgery by the German society for general and visceral surgery (DGAV), MIE was implemented in 2014. The surgical team performing MIE in our center was experienced in the technique prior to implementation. Since implementation, MIE represents the standard surgical treatment for patients with resectable esophageal cancer (EC) and cancer of the gastroesophageal junction (CGEJ) in our center, with 30–40 MIE and 5–10 hybrid MIE (open abdominal approach) performed in our center per year. We herein present the results of our institutional structured surgical training program in minimally invasive esophagectomy, including stratified patient selection and supervision. By comparing the LCs of an experienced surgeon with a trainee, we demonstrate that MIE can be taught and learned without impairing the oncological or perioperative outcome of patients during the learning process.

## Materials and methods

### Data collection

Data analysis included demographic details and patient characteristics, including sex, age, BMI, preexisting comorbidities, tumor stage, as well as perioperative data, including operation time, postoperative hospital stay, post-operative complications, 30- and 90-day mortality, and follow-up data (overall survival (OS)). Clavien-Dindo (CD) classification was applied to grade post-operative complications. Complications CD ≥ 3a were defined as major complications. Anastomotic leakage (AL) was defined as endoscopically and/or radiological (computed tomography or X-ray after oral intake of contrast) verified defect of the intestinal wall at the anastomotic site. The criteria for determining a Textbook Outcome (TO) were derived from Busweiler et al. [13], which included (a) clear resection margins (R0), (b) examination of at least 21 lymph nodes, (c) absence of postoperative complications categorized as Clavien-Dindo ≥ 3b, (d) no surgical re-interventions, (e) no unexpected admissions to ICU/IMC, (f) hospital stay under 21 days, (g) no hospital readmissions within 30 days post-discharge, and (h) absence of mortality within 30 days after the procedure. All data were prospectively collected in a database. Data analysis was performed retrospectively. Data collection was approved by the local ethics committee (EA2/212/23).

### Inclusion and exclusion criteria

**Fig. 1:**
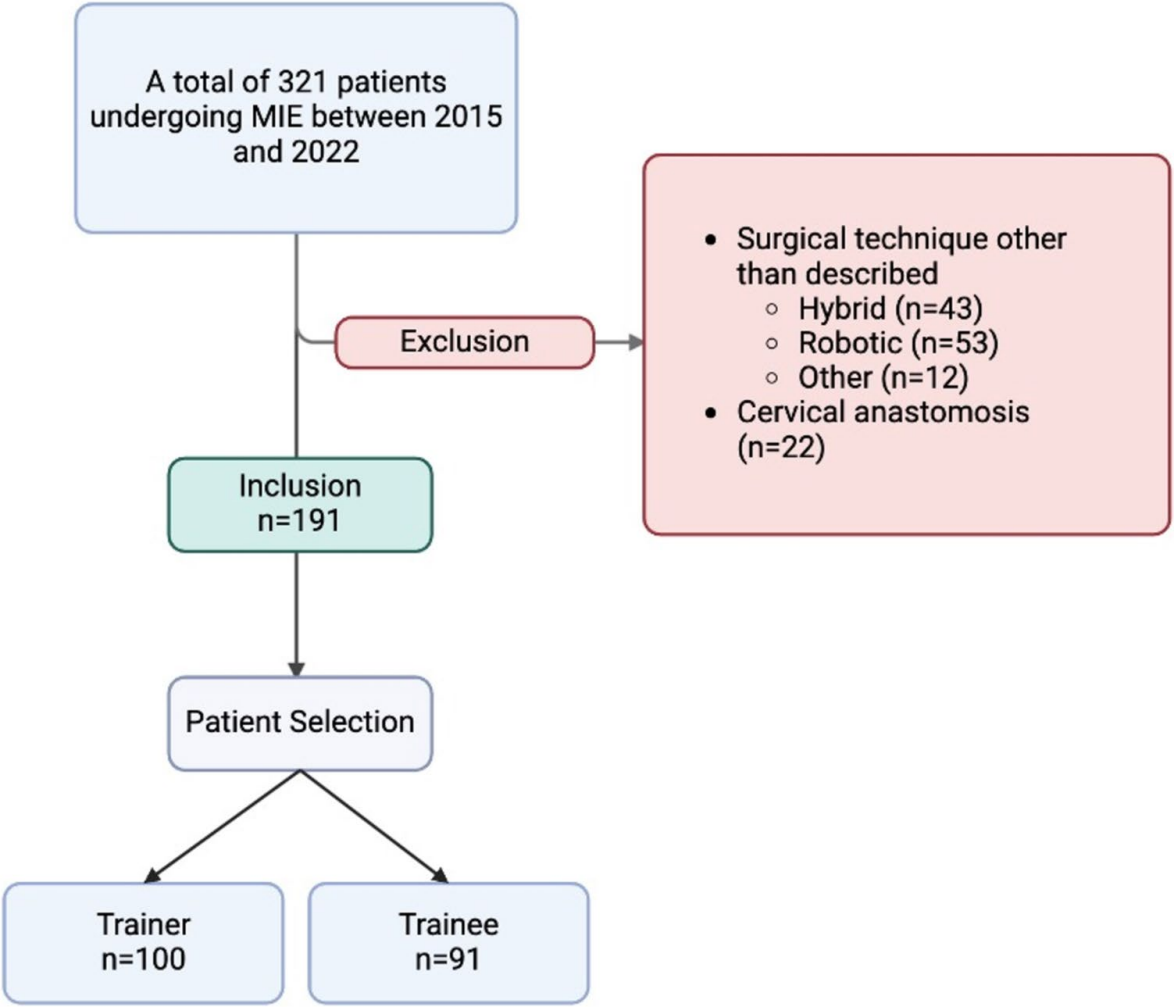
Inclusion, exclusion criteria, and patient selection for trainee and trainer. MIE–Minimally Invasive Esophagectomy. Created with BioRender.com

A total of 321 patients receiving MIE for EC between 2015 and 2022 in our center were included in this retrospective Analysis, while 191 patients met the inclusion criteria. Only patients with carcinomas of the esophagus or esophagogastric junction AEG I and II (cT1b-4a N0-3 M0), undergoing elective MIE in curative intention were included in the analysis. Patients undergoing MIE in palliative intention were excluded. Cases in which cervical anastomosis was performed or in which esophagectomy was performed with another minimally invasive approach than the described one (e.g., robotic-assisted, hybrid laparoscopic esophagectomy) were also excluded from the analysis (Fig. 1.). All patients underwent standardized staging diagnostics, including endoscopy and CT scan, and were discussed in our institutional multidisciplinary tumor board prior to surgery. If recommended by the tumor board and according to the current national guideline, patients received neoadjuvant chemo- and/or radiotherapy prior to surgery [10].

### Surgical procedure and perioperative management

MIE was performed as previously described [11]. In short, laparoscopic gastric mobilization and systematic lymphadenectomy (LAD) was followed by transthoracic minimally invasive esophagectomy with 2-field LAD in the sense of an Ivor Lewis procedure. To restore enteral continuity, gastric pull-up with either circular stapled end-to-side anastomosis or linear stapled side-to-side anastomosis was performed. Circular stapled anastomosis was secured by handsewn V-Loc sutures. The integrity of the anastomosis was confirmed intraoperatively via endoscopy and a nasogastric tube was placed under view. A chest tube was routinely placed. After surgery, all patients were admitted to our specialized intensive care unit for at least 2 days with immediate start of oral fluid intake. Nasogastric tubes were removed on the second day. Subsequently, enteral nutrition was started and adapted according to patient tolerance.

### Surgeons prior experience

Our surgical department comprises two campuses and is accredited as a tertiary referral center for gastroesophageal surgery by the German society for general and visceral surgery (DGAV). MIE was implemented in 2014 by our surgical team. Since the implementation, approximately 40–50 esophageal resections are performed each year, nowadays including 10–15 MIEs, 5–10 hybrid MIEs, and 25–30 robotic-assisted resections. MIE and more recently RAMIE represent the standard curative surgical treatment for patients with EC in our center. Both surgeons, trainer as well as trainee, were experienced specialists for general and visceral surgery with prior expertise in upper GI surgery (Table 1.).

**Table 1:** Prior surgical experience of the observed surgeons at the beginning of the observed period (2017)

	Surgeon 1–Trainer	Surgeon 2–Trainee
Time since specialization in general surgery (years)	8	5
Time since specialization in visceral surgery (years)	6	1
Prior MI bariatric surgeries^1^ (count)	210	350
Prior gastrectomy performed (count)	120	70
Prior esophagectomy performed (count)	90	0

MI bariatric surgeries included sleeve gastrectomy, gastric bypass, and single anastomosis duodenal-ileal bypass with sleeve (SADI-S). All counts are given rounded. MI—minimally invasive

### Teaching concept and patient selection during learning period

The training program aims to achieve surgical autonomy in MIE for advanced upper GI surgeons with prior experience in gastric and bariatric surgery. Surgical autonomy was considered achieved when the trainee was able to independently perform complete surgical procedure. The criteria for autonomy required that the trainee's outcomes for textbook outcomes (TO), complication rates (including anastomotic leak (AL) and pneumonia), and perioperative morbidity consistently matched those of the trainer as well as international benchmarks. The trainee was considered autonomous when they were able to uphold this standard without any concessions in terms of operative time. This milestone was a key indicator of the trainee’s readiness to transition from supervised operations to performing surgeries independently, ensuring they met both national and international standards of surgical care.

**Fig. 2:**
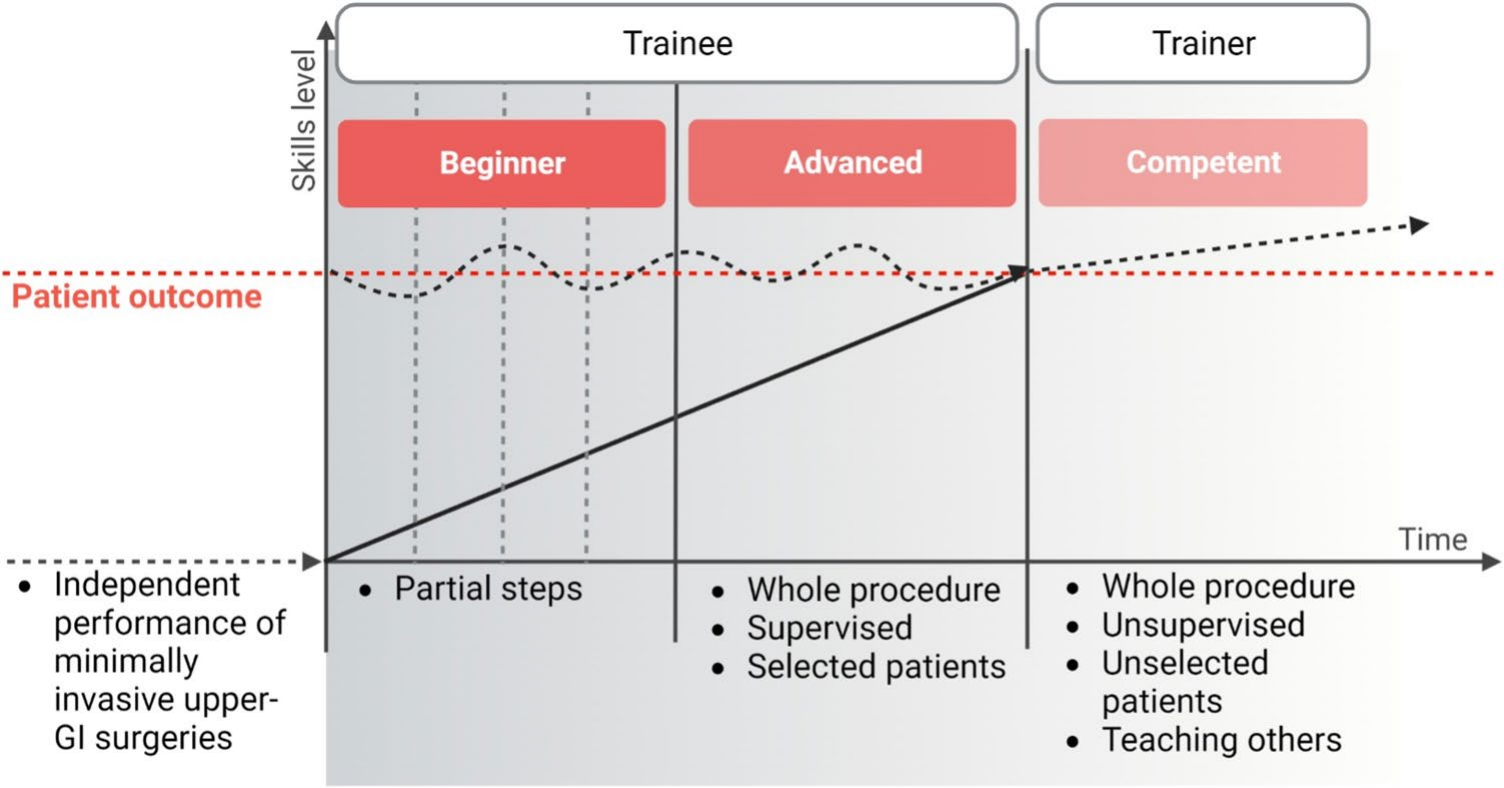
Applied institutional teaching concept. Aimed patient outcome (red dashed line) during the trainee´s LCs emphasized the expected outcome, regularly achieved by the competent surgeon. Created with BioRender.com

The study covers the training program of one trainer surgeon and one trainee surgeon. The trainees underwent a standardized program at our institution outlined in Fig. 2. All procedures included were performed by both surgeons (trainer and trainee) together. All surgeries were additionally assisted by an upper GI fellow. This pathway includes prior training with a laparoscopic trainer/simulator, followed by bariatric surgery, minimally invasive gastrectomy (MIC), and then minimally invasive esophagectomy (MIE). This sequence does not vary for trainees. In the beginning of the MIE training period, the trainee performed individual sub-steps of the procedure. With growing experience, selected cases were performed by the trainee under supervision and assistance from the trainer (Fig. 2). Patient selection was made at the trainer’s discretion based on preoperative patient characteristics affecting technical complexity and perioperative outcome. Those criteria included normal BMI, preoperative tumor size < T2, low ASA score in particular the absence of preexisting cardiac disease and absence of diabetes. Due to this selection bias, risk adjustment was performed in LC analysis for exclusion of any bias.

### Defining the learning curve (LC) for minimally invasive esophagectomy for esophageal cancer

The training program is designed to enable advanced upper GI surgeons to achieve surgical autonomy in minimally invasive esophagectomy (MIE), while maintaining a consistent standard of care and stable rates of complications. The primary objective of the program is to reduce morbidity and mortality which are generally associated with the learning curve. As such, essential outcome metrics like textbook outcomes, anastomotic leak (AL), and morbidity should not change drastically if the program is efficient, operative time functions as a surrogate parameter in this case. To calculate the LC of the two observed surgeons, cumulative sum (CUSUM) was applied. CUSUM is a statistical tool used to monitor small shifts in the mean of a process, effectively identifying deviations from expected performance standards. CUSUM uses a cumulative sum of deviations from a predefined standard to track a surgeon’s performance over time. This approach has been applied especially during training of a new surgeon or implementation of a new surgical technique. Herein, we used RA-CUSUM analysis. Risk adjustment is to define a surgeon’s learning curve according to case mix and the likelihood of adverse events. Due to the application of risk adjustment, RA-CUSUM analysis is less susceptible to outliers or selection bias which helps to identify underlying trends. In our analysis, performance measures included operation times (OT), length of stay (LOS), Nights on ICU, and lymph node yield (LN Yield). Risk-adjusted RA-CUSUM analysis was also performed to assess the LC in terms of anastomotic leakage (AL), major complications (MC, Clavien Dindo ≥ 3a), and achieved textbook outcomes (TO). Risk adjustment was performed based on age, sex, BMI, preoperative tumor size < T2, ASA score and UICC Score, and absence of preexisting heart disease or diabetes. Additional demographic information was not included in either analysis as there were no significant differences between the groups and no significance in terms of target parameters (MC, TO, and AL). Linear and logistic regression models based on these parameters were established and log-transformed data were included in RA-CUSUM analysis.

### Further statistical analysis

To compare datasets regarding patient characteristics and outcomes between trainee and trainer, statistical analyses were conducted using Student’s t test for continuous variables that were confirmed to follow a normal distribution. The normality of continuous variables was validated using the Shapiro–Wilk test. For non-normally distributed variables, non-parametric tests such as the Mann–Whitney U test were applied. Categorical data were compared using the χ^2^ test. Multivariate analysis was performed using a nominal logistic regression model. Statistical significance was defined as a p value below 0.05. Survival analysis for overall survival (OS) and disease-free survival (DFS) was performed using the Cox proportional hazards model. All analyses were performed using JMP Pro®, Version 16. SAS Institute Inc., Cary, NC, 1989–2021.

## Results

### Patient characteristics and histopathology

The comprehensive overview of preoperative patient characteristics and histopathological findings is presented in Table 2. An examination of key demographic variables across the groups, which includes age, gender distribution, and preoperative comorbidities, revealed no statistically significant differences. Similarly, our analysis of histopathological data, encompassing parameters such as tumor stage, nodal involvement, and tumor differentiation, indicated a lack of substantial variations among the groups apart from advanced tumor size (pT4; Trainer vs. Trainee, 0.00% vs. 6.59% (*p* = 0.027)). This was accounted for in subsequent LC analysis by risk adjustment. These cases were performed at the end of the learning curve and may reflect the increased implementation of the laparoscopic approach in locally progressive disease.

**Table 2:** Clinical and Histopathological characteristics of Patients after MIE for EC or cancer of the GEJ

Characteristics	Surgeon 1–Trainer n = 100	Surgeon 2–Trainee n = 91	*p*
Male sex, n (%)	83 (83.00)	75 (81.21)	0.762
Mean age at resection, years (± SD)	63.38 (± 9.98)	65.25 (± 9.21)	0.178
Mean BMI, kg/m^2^ (± SD)	25.72 (± 5.38)	25.90 (± 4.90)	0.808
Comorbidities			
Diabetes, n (%)	12 (12.00)	16 (17.58)	0.478
Heart disease, n (%)	27 (27.00)	27 (29.67)	0.682
Pulmonary disease, n (%)	14 (14.00)	17 (18.68)	0.381
Liver disease, n (%)	5 (5.00)	4 (4.40)	0.844
ASA physical status, n (%)			
I	5 (5.00)	5 (5.49)	0.773
II	46 (46.00)	35 (38.46)	
III	48 (48.00)	50 (54.95)	
IV	1 (1.00)	1 (1.10)	
Preoperative therapy, n (%)			
Chemotherapy	83 (83.00)	79 (86.81)	0.462
Radiochemotherapy	45 (45.00)	33 (36.26)	0.219
Tumor location, n (%)			0.641
Esophagus	56 (56.00)	54 (59.34)	
Gastroesophageal junction	44 (44.00)	37 (40.66)	
Tumor size, n (%)			0.027
pT0	27 (27.00)	21 (23.08)	
pT1	23 (23.00)	15 (16.48)	
pT2	19 (19.00)	15 (16.48)	
pT3	31 (31.00)	34 (37.36)	
pT4	0 (0)	6 (6.59)	
Nodal status, n (%)			0.450
pN0	64 (64.00)	49 (53.85)	
pN1	20 (20.00)	20 (21.98)	
pN2	10 (10.00)	15 (16.48)	
pN3	6 (6.00)	7 (7.69)	
UICC stage, n (%)			0.184
I	43 (43.00)	42(46.15)	
II	17 (17.00)	7 (7.69)	
III	33 (33.00)	31 (34.07)	
IV	7 (7.00)	11 (12.09)	
Histologic type, n (%)			0.700
Adenocarcinoma	70 (70.00)	66 (72.53)	
Squamous cell carcinoma	30 (30.00)	25 (27.47)	
Tumor grading (G), n (%)			0.311
G1	9 (9.00)	8 (8.79)	
G2	52 (52.00)	45 (49.45)	
G3	35 (35.00)	28 (30.77)	

*BMI* body-mass index, *ASA* American society of anesthesiology, *UICC* Union for international cancer control

### Postoperative outcome

Perioperative patient outcomes in both groups are summarized in Table 3. Our investigation reveals a comparable median duration of resection (Trainer vs. Trainee, 404.40 min vs. 383.07 min (*p* = 0.085)). Similarly, there was no significant difference in terms of the median count of lymph nodes extracted (Trainer vs. Trainee, 29.5 [25–37.75] vs. 32 [26–39] (*p* = 0.0789)). The observed trends toward shorter operation times and higher LN yields may reflect an overall continuous improvement of the general technique during the observational period of 7 years. The occurrence of positive resection margins depicted similar trends (Trainer vs. Trainee, 6.00% vs. 3.30% (*p* = 0.281)), as did the need for intraoperative red blood cell transfusions (Trainer vs. Trainee, 11.00% vs. 6.59% (*p* = 0.281)).

**Table 3:** Perioperative parameters of patients who underwent MIE for EC or cancer of the GEJ

Characteristics	Surgeon 1 – Trainer n = 100	Surgeon 2 – Trainee n = 91	*p*
Median duration of resection (± SD), minutes	404.40 (± 75.80)	383.07 (± 84.88)	0.085
Median number of lymph nodes removed, median [IQR]	9.5 [25–37.75]	32 [26–39]	0.079
Positive resection margins, n (%)	6 (6.00)	3 (3.30)	0.373
Need for intraoperative RBC transfusions, n (%)	11 (11.00)	6 (6.59)	0.281
Intraoperative blood loss, ml (± SD)	131.60 (± 223.14)	88.91 (± 117.53)	0.120
Anastomosis			
Circular 25 mm	49 (49.00)	16 (17.98)	< 0.001
Circular 29 mm	40 (40.00)	43 (48.31)	
Other	11 (11.00)	30 (33.71)	
Major complications, n (%)	25 (25.00)	13 (14.29)	0.062
Complications, n (%)	41 (41.00)	39 (42.86)	0.800
Pulmonary, n (%)	36 (36.00)	34 (37.36)	0.864
Pneumonia, n (%)	27 (27.00)	18 (19.78)	0.239
Cardiovascular, n (%)	6 (6.00)	3 (3.30)	0.373
Anastomotic leak, n (%)	10 (10.00)	7 (7.69)	0.575
Duration of ICU stay (days), median [IQR]	3 [2.25–6.75]	3 [2–5]	0.036
Median duration of hospital stay (days), median [IQR]	6.5 [13–31.5]	16 [12–22]	0.036
30-day mortality, n (%)	1 (1.00)	0 (0.00)	0.256
Textbook outcome, n (%)	27 (27.00)	37 (40.66)	0.046

*ICU–Intensive care unit; RBC, red blood cells; SD, Standard deviation*

Regarding complications, the incidence of major complications showed no significant difference under the care of the trainee (Trainer vs. Trainee, 25.00% vs. 14.29% (*p* = 0.062)). There was no difference in terms of pneumonia (Trainer vs. Trainee, 27.00% vs. 19.78% (*p* = 0.239)), cardiovascular complications (Trainer vs. Trainee, 6.00% vs. 3.30% (*p* = 0.373)), or anastomotic leak (Trainer vs. Trainee, 10.00% vs. 7.69% (*p* = 0.575)). Despite similar rates of complications, a significantly shorter ICU stay was seen in patients with the operation performed by the trainee (Trainer vs. Trainee, 3 [2.25–6.75] days vs. 3 [2–5] days (*p* = 0.0359)), and a trend toward a shorter duration of hospital stay (Trainer vs. Trainee, 16.5 [13–31.5] days vs. 16 [12–22] days (*p* = 0.0361)). These differences again are accounted for by the continuous improvement of postoperative patient management supporting enhanced recovery.

No difference was seen in 30-day mortality rates (Trainer vs. Trainee, 1.15% vs. 0.00% (*p* = 0.256)). Finally, the occurrence of textbook outcomes was significantly higher in the trainee cohort (Trainer vs. Trainee, 27.00% vs. 40.66% (*p* = 0.046)).

### Learning curves for trainee and trainer

**Fig. 3:**
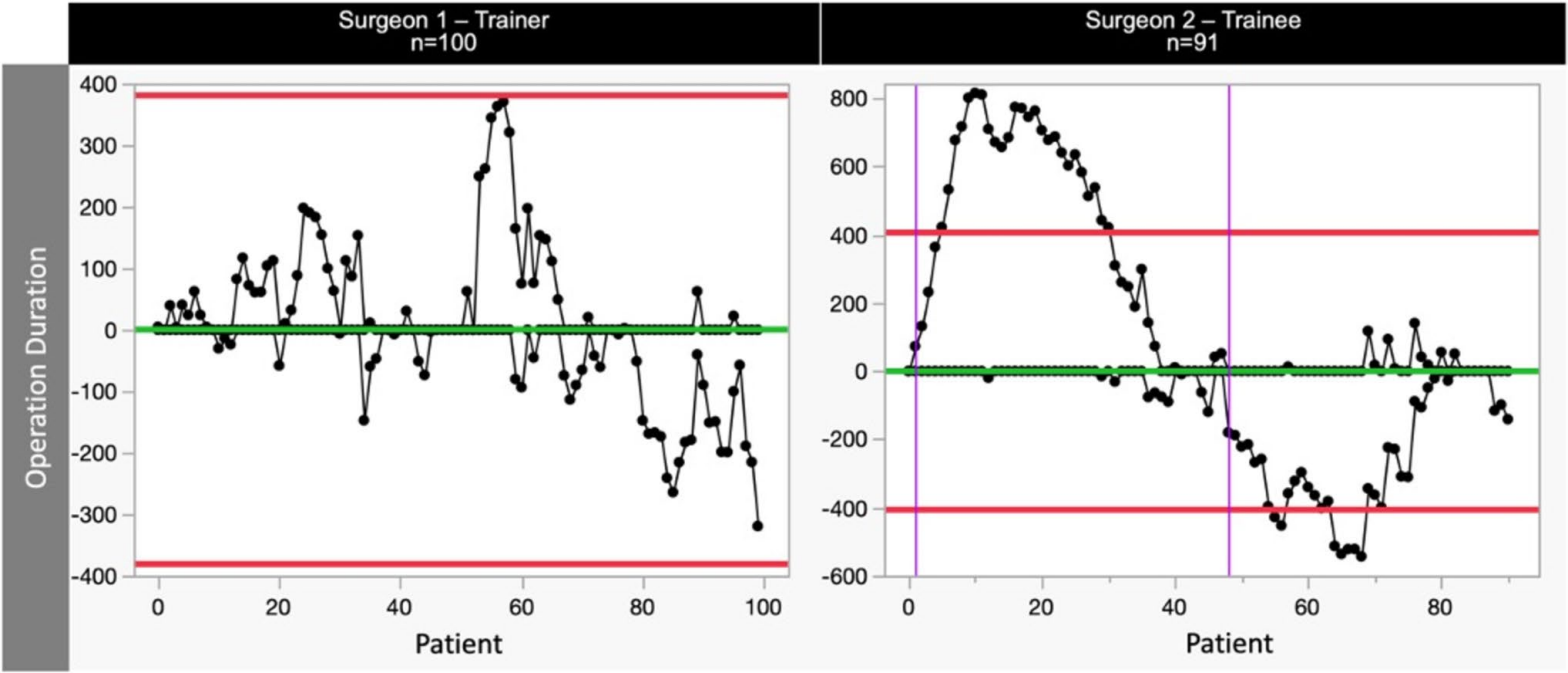
Two-sided RA-CUSUM learning curves of perioperative parameters for trainer (left) and trainee (right). Red horizontal lines reflect the upper (UCL) and lower (LCL) control limits and purple vertical lines represent a shift in means

**Fig. 4:**
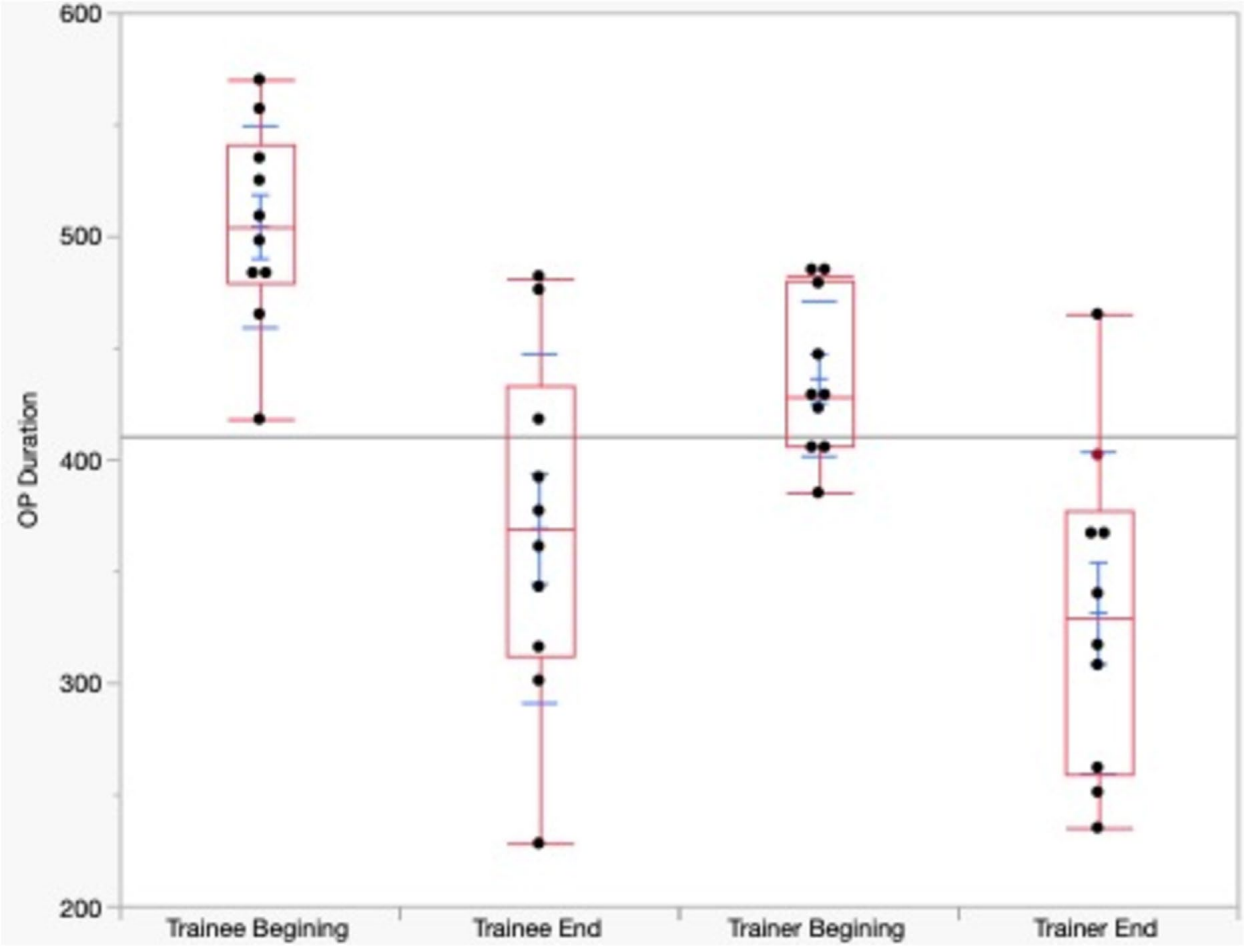
Improvement in terms of operating time in both the trainee and trainer during the observation period in the first 10 cases vs the last 10 cases

A comprehensive analysis of the RA-CUSUM results reveals intriguing patterns in operation duration for both the trainer and trainee. Initially, the trainee exhibited longer operation times compared to the trainer, as shown by the higher consecutive values on the CUSUM chart (Fig. 3). However, the mean operating time did not differ between the trainee and trainer across the entire cohorts (Trainer 404.40 (± 75.80) vs. Trainee 383.07 (± 84.88), *p* = 0.085). The trainer, despite maintaining a steady operation duration at the outset, displayed enhanced efficiency in terms of OT after an additional 80 cases (Fig. 3). A comparative analysis between the initial and the final outcomes (initial 10 vs. final 10 cases) (Fig. 4) showed that both the operative times of the trainee and trainer significantly decreased during the training period. The OT of the trainer decreased from 436,4(± 34,77) to 331,4(± 72,00) (*p* < 0.001), while the OT of the trainee decreased from 504,4 (± 45,20) to 369,3(± 78,20) (*p* < 0.001).

**Fig. 5:**
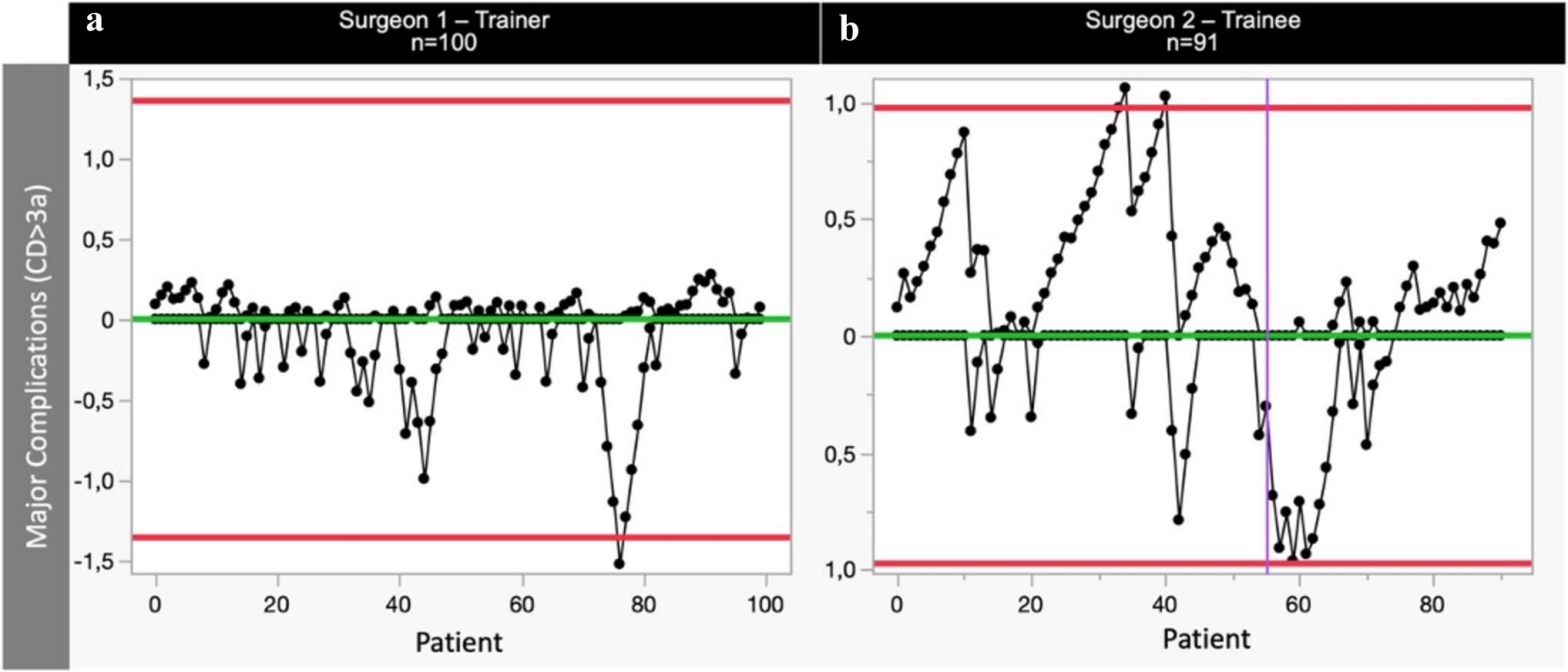
One-sided RA-CUSUM learning curves of major complications (MC) for trainer **a** and trainee **b**. Red horizontal lines reflect the upper control limit (UCL) and lower control limit (LCL) and purple vertical lines represent a shift in means

**Fig. 6:**
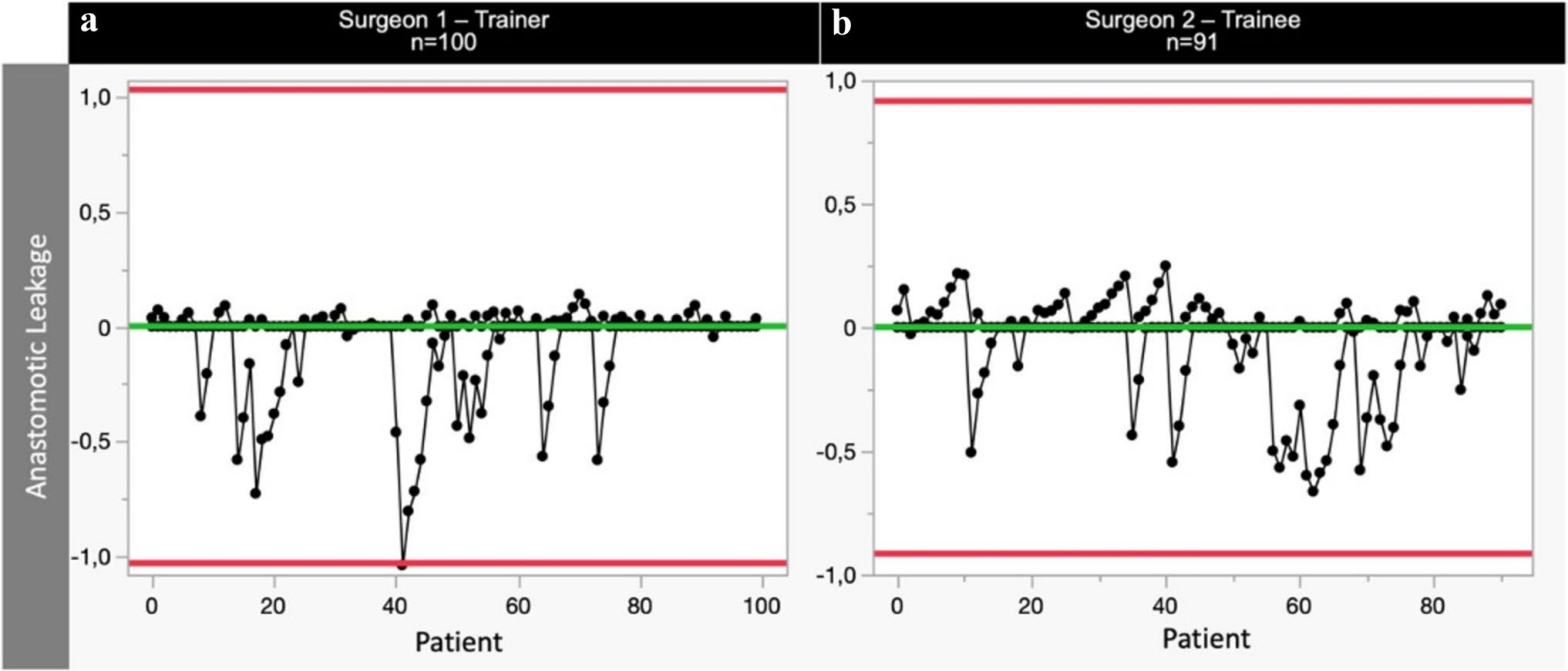
One-sided RA-CUSUM learning curves of anastomotic leakage (AL) for trainer **a** and trainee **b**. Red horizontal lines reflect the upper control limit (UCL) and lower control limit (LCL) and purple vertical lines represent a shift in means

The RA-CUSUM analysis for Major Complications (Fig. 5) revealed a distinctive LC with respect to MC rates. Throughout the learning period, the trainee consistently achieved MC rates comparable to those of the trainer. Following risk adjustment, we observed a noteworthy improvement in the trainee's MC incidence after the completion of 55 consecutive cases. This may reflect the influence of patient selection during the beginning of the learning curve. However, a clear LC was evident in technical operative skills after 45 cases (operative time, Fig. 3). Interestingly, no LC was seen in terms of AL with consistent rates seen in both the trainee and trainer cohort (Fig. 6). Both the trainer and trainee consistently maintained comparable and stable AL rates of 10.0% and 7.7%, respectively (*p* = 0.575). Of note, the anastomotic technique was adjusted during the teaching period from a 25 mm to a 29 mm circular anastomosis, thereby potentially influencing the AL rates in the latter period positively [11].

**Fig. 7:**
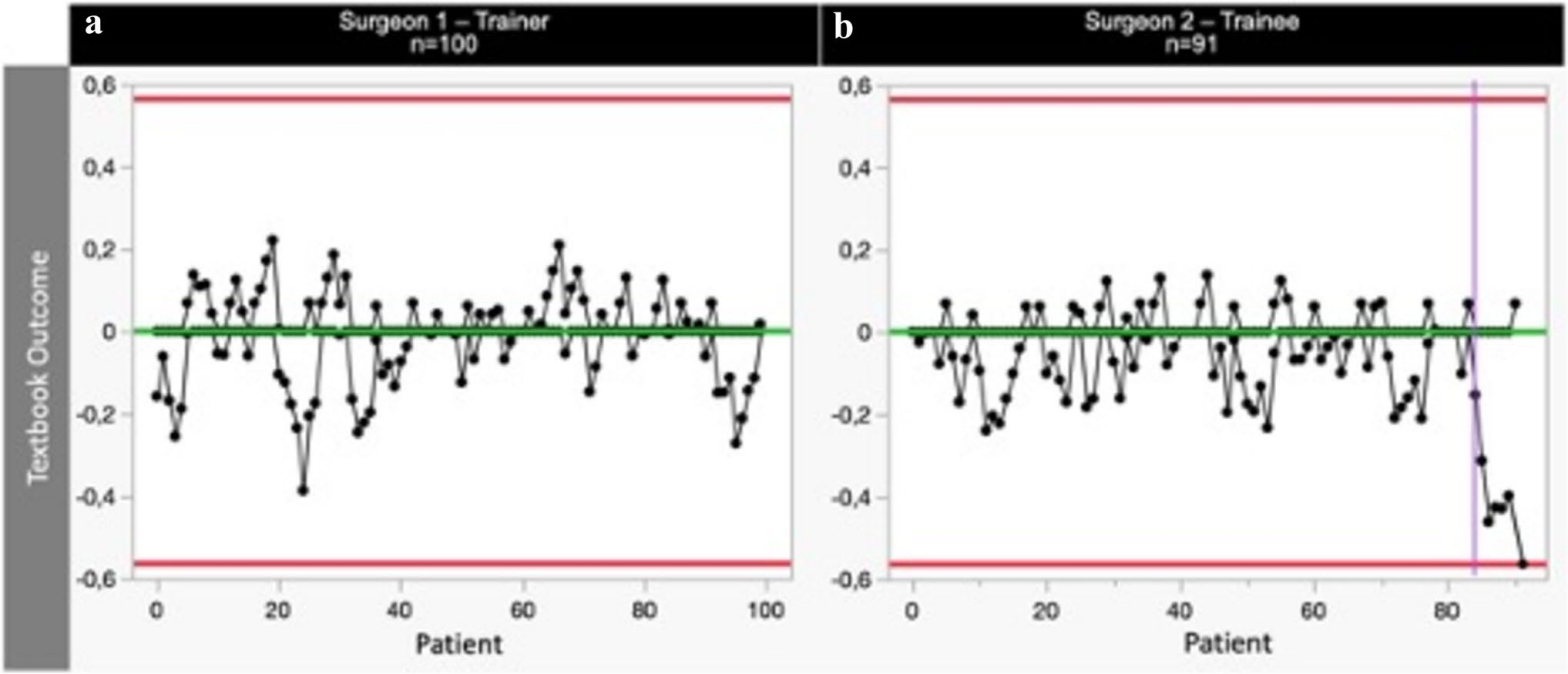
One-sided RA-CUSUM learning curves of textbook outcome (TO) for trainer **a** and trainee **b**. Red horizontal lines reflect the upper control limit (UCL) and lower control limit (LCL) and purple vertical lines represent a shift in means

Examining the composite parameter TO (Fig. 7), our investigation also revealed a learning curve after 83 cases. The rate of TO achievement by the trainee never dropped below that of the trainer. Finally, the occurrence of textbook outcomes was significantly higher in the trainee cohort overall (Trainer vs. Trainee, 27.00% vs. 40.66% (*p* = 0.046)). It is apparent that this parameter encompasses multifaceted aspects of surgical management and is considerably influenced by factors beyond the realm of operative performance only.

### Survival

**Fig. 8:**
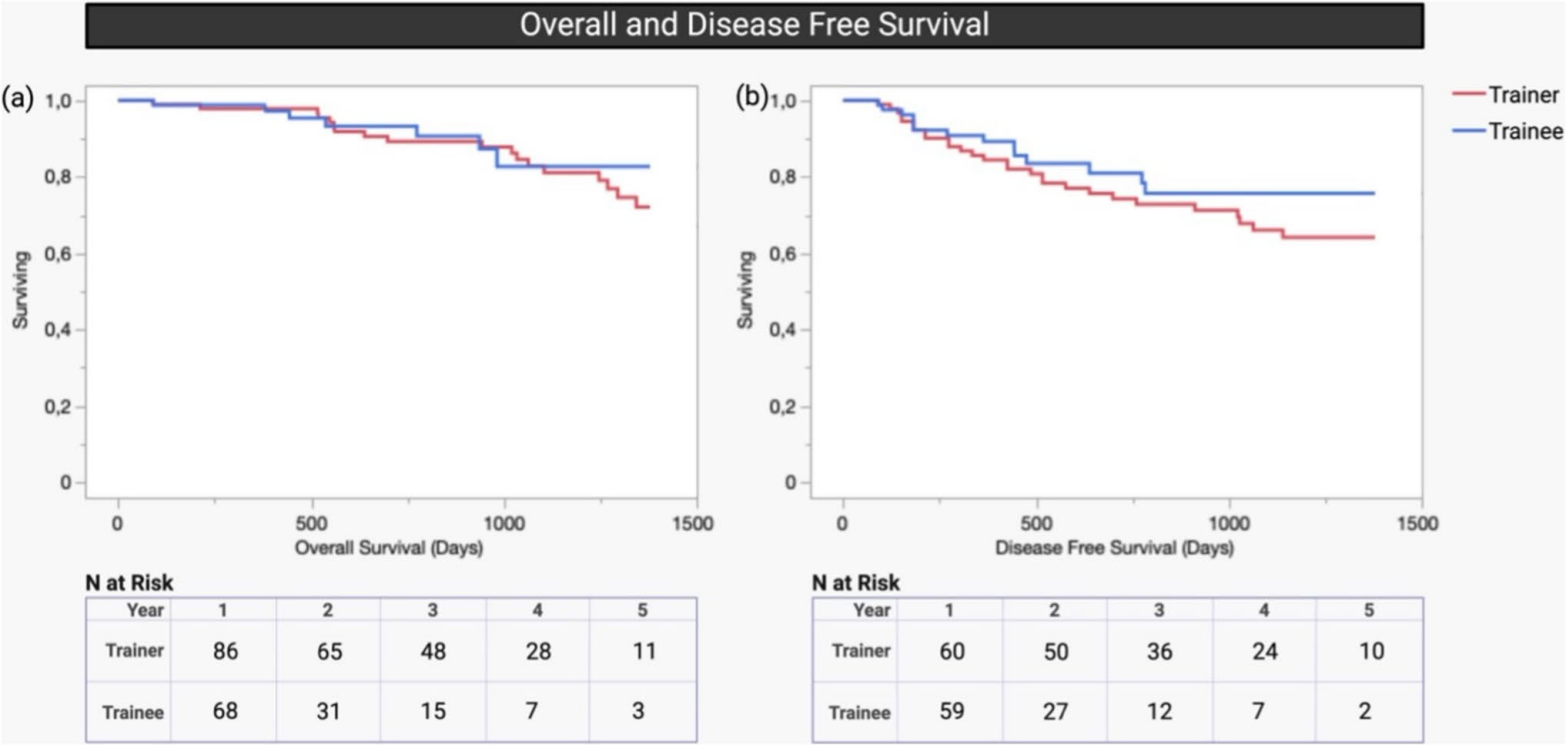
Kaplan–Meier survival curve illustrating comparable **a** overall survival (1-year OS: Trainer = 97.84% vs. Trainee 98.80%; 3-year OS: Trainer = 82.88% vs. Trainee = 82.71%) (p = 0.436)) and **b** disease-free survival (1-year DFS: Trainer = 84.42% vs. Trainee 90.81%, 3-year DFS: Trainer = 66.03% vs. Trainee = 75.67%) (p = 0.436)). The mean follow-up duration was 1094 (+ 580) days in the trainer group and 641(+ 484) days in the trainee group

To ensure an adequate follow-up time of at least 2 years, only patients operated between 2015 and 2020 were included in the survival analysis. Survival analysis comprised a total of 97 patients, 66 in the Trainer group and 31 in the Trainee group. The study encompassed a mean follow-up duration of 1094 (+ 580) days in the trainer group and 641(+ 484) days in the trainee group. The 1-year overall survival rates stood at 97.84% for the Trainer group and 98.80% for the Trainee group, while the 3-year survival rates were 82.88% and 82.71%, respectively (*p* = 0.436). The 1-year disease-free survival rates were 84.42% and 90.81%, while the 3-year survival rates were 66.03% and 75.67%, respectively (*p* = 0.305). Notably, no statistically significant distinctions emerged in terms of survival rates, highlighting a comparable outcome between the Trainer and Trainee groups (Fig. 8.). This indicated that the learning curve did not compromise oncological or surgical safety of the operation.

## Discussion

Minimally Invasive Esophagectomy (MIE) presents significant learning challenges but offers key benefits for esophageal and gastroesophageal junction (GEJ) cancer patients. Despite the learning curve (LC) inherent to even seasoned surgeons, prioritizing patient safety and optimal outcomes remains paramount. Structured training, selective patient assignment, and ongoing quality assurance are essential to enhance teaching effectiveness and maintain brief LCs. Our institutional review of 191 MIE procedures (2015–2022), including a trainee working with an experienced surgeon, showed no notable difference in postoperative complications, overall survival or TO throughout the LC, demonstrating the viability of our educational strategy.

Operating time is a common performance metric used in LC evaluation. In the literature, operative durations ranging from 237 to 443 min have been described for MIE [12, 13]. The reported learning curve in previous studies ranges from 80 to 119 [12–15]. In our study, the trainee completed the learning curve within 45 cases with a mean operating time of 383.07 (± 84.88). While one pervious study reports similar values [15], this is generally shorter than the LC reported in the literature [12–15]. Of note, during the observed period, 51 patients with EC underwent hybrid minimally invasive esophagectomy (open technique for abdominal part) and 54 underwent robotic-MIE (RAMIE) by the same surgical team, including trainer and trainee, adding further experience [15]. These cases were not accounted for in our study and may contribute to the comparably shorter learning curve observed in terms of operation time compared to the literature. Interestingly, a trend toward a further improvement of the operating time could also be seen for the trainer after 63 cases despite proficiency. The continued refinement in operating time by the trainer possibly underscores ongoing skill enhancement driven by increased experience and familiarity with the procedure.

Research by Valsangkar et al. suggests that neither short nor intermediate lengths of surgery time consistently predict postoperative results following Ivor Lewis esophagectomy [16]. This finding casts doubt on the reliability of operative time as a solitary metric for gauging the learning curve or shifts in performance levels. Interestingly, the learning curve for operative time (most specifically representing technical proficiency of the surgeon) is considerably shorter than the learning curve for other perioperative parameters observed in our study. The longer LC observed in MC or TO compared to operative time underscores the intricate interplay between mastering the technical skills required and the multifaceted dimensions of surgical proficiency. This prolonged trajectory signifies the complex nature of acquiring surgical judgment and ability to provide the comprehensive perioperative care involved in complication management.

Using the trainer´s surgical results as the outcome target to be achieved, we observed that the learning curve was achieved after 55 cases in terms of major complications after risk adjustment. The results of our study partly differ from the experiences of other surgical centers, which analyzed LCs for MIE during the implementation. In contrast with our observations, two multicenter studies by van Workum et al. in 2019 and Claassen et al. in 2022 showed increased morbidity during MIE LCs [12, 13]. These studies report the achievement of the learning curve in terms of major complications after 34 and 119 cases, respectively, indicating a longer learning curve than that seen in our cohort [12, 13, 15]. An explanation for this might be that the mentioned studies analyzed the LC during newly implementation of MIE in different surgical centers. In contrast, our study analyzed the LC of a learning surgeon in a center in which MIE was already an established procedure. Furthermore, Claassen et al. included patients with tumors localized in the middle third of the esophagus, which could also contribute to the discrepant LC results. In contrast to other authors, we excluded patients with cervical anastomosis in our study. Finally, the additional experience of the surgeons in Hybrid-MIE and RAMIE may have augmented the learning experience [17, 18].

In our study, we did not observe a significant LC for anastomotic leakage. Van Workum et al*.* and Claassen et al*.* described the number of performed cases of 119 and 131 to achieve AL rates of 8% and 14%, respectively [12, 13]. Considering the number of cases performed during the time observed in our study (100 cases performed by the trainer, 91 cases performed by the trainee), we cannot confirm these values. However, we observed stable and comparable AL rates for both trainer and trainee, respectively. Using the values of the trainer as a benchmark, AL rates of the trainee remained in the target range throughout the study. The style of anastomosis was adapted throughout the learning period from 25 to 29 mm circular anastomosis. A 29 mm esophagogastrostomy has been linked to reduced rates of AL [11]. While we saw a trend toward reduced AL rates after 60 cases, this trend was not sufficient to result in a shift in means detected by RA-CUSUM analysis.

Despite the evolving proficiency gain demonstrated in other domains, TO also remained resilient to the characteristic learning curve trajectory observed in this study. In contrast, Claassen et al. report a learning curve in terms of TO of 46 cases [13]. The rate of TO consistently achieved by the trainee during the whole learning period was in line with those described in the literature. A rate of TO of 30.7% was previously described in a large international study by the Oesophago-Gastric Anastomotic Audit (OGAA) Collaborative [19–21]. Furthermore, the rate of TO achieved by the trainee did not fall below that of the trainer at any point throughout the learning curve. A further improvement of TO incidence was observed after the performance of 83 consecutive cases by the trainee. TO as composite measure of surgical outcomes defines the best possible outcome of a surgical procedure [21]. Our results suggest that the intricacies of patient care, post-operative recovery, and potential medical interventions contribute significantly to this parameter [21]. This underscores the complex, multifactorial nature of TO as a performance metric, and highlights the non-operative skill acquisition required by surgeons for optimal complication management.

Our study's methodology involved a highly selective patient screening process, where patients with a tumor size > T2, ASA > 2 score, prior cardiac disease or diabetes, or a BMI > 30 were identified as higher risk and were therefore more likely to have their resections performed by the experienced surgeon only. While this approach minimized the risks associated with surgical training, it introduced an unavoidable selection bias in our results. This bias is particularly evident in the significantly higher numbers of postoperative days in the ICU and longer hospital stays (LOS) for patients operated by the trainer, likely because more complex cases were indeed handled by more experienced hands. Additionally, due to the long observational period in our study, this also reflects the adoption of ERAS principles throughout our study period. During the earlier phases of our study period, there was a more cautious postoperative approach, particularly in complex cases handled by trainers, leading to longer ICU stays. Over time, our institution gradually integrated Enhanced Recovery After Surgery (ERAS) protocols, which include immediate mobilization, intensive conservative respiratory management, and enhanced physiotherapy.

Moreover, the inclusion of only one trainee in our analysis significantly impacts the generalizability of our findings. The insights gained are inherently limited by this single-trainee framework, restricting the applicability of our results to broader educational settings or diverse surgical teams. This aspect of our study design should prompt caution when extrapolating our findings to other institutions or training programs.

In addition, esophagectomies involving other related procedures were systematically excluded from our study, potentially introducing additional bias. This exclusion might skew the complexity and outcomes associated with standard esophagectomies and could mask the true challenges encountered in surgeries that involve more extensive operative scopes.

Risk adjustment was implemented in our RA-CUSUM analysis to mitigate some of the biases; however, this was not extended to the comparison of event distributions across the entire cohort (Table 1 and Table 2). The study’s case volume, while aligned with the annual benchmark for German surgical centers [16], remains limited, and therefore, the absence of significant differences in major complications rates should not be over-interpreted. The small sample size could underpower statistical tests, potentially overlooking real differences or misinterpreting the surgical outcomes.

These limitations underscore the need for further studies with larger, more diverse cohorts and a variety of training setups to validate and possibly extend our findings.

## Conclusions

Based on the results of the analysis of the data of 191 patients undergoing MIE in our center between 2015 and 2022, we state that MIE can be trained and learned by experienced upper gastrointestinal surgeons without compromising oncological and surgical outcome in patients with resectable EC/CGJ. Structured surgical training facilitates consistent perioperative outcomes throughout the learning period. This study emphasizes the importance of structured training programs and continued risk assessment for enhanced patient care.

## Data Availability

Data available from authors upon request.
